# The greatest air quality experiment ever: Policy suggestions from the COVID-19 lockdown in twelve European cities

**DOI:** 10.1371/journal.pone.0277428

**Published:** 2022-11-30

**Authors:** Marialuisa Volta, Umberto Giostra, Giorgio Guariso, Jose Baldasano, Martin Lutz, Andreas Kerschbaumer, Annette Rauterberg-Wulff, Francisco Ferreira, Luìsa Mendes, Joana Monjardino, Nicolas Moussiopοulos, Christos Vlachokostas, Peter Viaene, Janssen Stijn, Enrico Turrini, Elena De Angelis, Claudio Carnevale, Martin L. Williams, Michela Maione

**Affiliations:** 1 Dipartimento di Ingegneria Meccanica e Civile, Università di Brescia, Brescia, Italy; 2 Dipartimento di Scienze Pure e Applicate, Università di Urbino Carlo Bo, Urbino, Italy; 3 Dipartimento di Elettronica, Informazione e Bioingegneria, Politecnico di Milano, Milano, Italy; 4 Centro Nacional de Supercomputación, Universitat Politècnica de Catalunya, Barcelona, Spain; 5 Senatsverwaltung für Umwelt, Mobilität, Verbraucher-und Klimaschutz, Berlin, Germany; 6 Departamento de Ciências e Engenharia do Ambiente, Faculdade de Ciencias e Tecnologia Universidade Nova de Lisboa, Caparica, Portugal; 7 Aristoteleio Panepistemio Thessalonikes, Thessalonike, Greece; 8 VITO, Vision on Technology, Mol, Belgium; 9 Environmental Research Group, Imperial College, London, United Kingdom; 10 Istituto di Scienze dell’Atmosfera e del Clima, Consiglio Nazionale delle Ricerche, Bologna, Italy; Oswaldo Cruz Foundation, BRAZIL

## Abstract

COVID-19 (Coronavirus disease 2019) hit Europe in January 2020. By March, Europe was the active centre of the pandemic. As a result, widespread "lockdown" measures were enforced across the various European countries, even if to a different extent. Such actions caused a dramatic reduction, especially in road traffic. This event can be considered the most significant experiment ever conducted in Europe to assess the impact of a massive switch-off of atmospheric pollutant sources. In this study, we focus on in situ concentration data of the main atmospheric pollutants measured in twelve European cities, characterized by different climatology, emission sources, and strengths. We propose a methodology for the fair comparison of the impact of lockdown measures considering the non-stationarity of meteorological conditions and emissions, which are progressively declining due to the adoption of stricter air quality measures. The analysis of these unmatched circumstances allowed us to estimate the impact of a nearly zero-emission urban transport scenario on air quality in 12 European cities. The clearest result, common to all the cities, is that a dramatic traffic reduction effectively reduces NO_2_ concentrations. In contrast, each city’s PM and ozone concentrations can respond differently to the same type of emission reduction measure. From the policy point of view, these findings suggest that measures targeting urban traffic alone may not be the only effective option for improving air quality in cities.

## Introduction

The Coronavirus pandemic, known as COVID-19 (Coronavirus disease 2019), was firstly described in December 2019 in the Hubei region in China and quickly reached Europe, with the first reported case in Germany by mid-January 2020 [1]. However, subsequent studies have suggested that the virus has been circulating in Europe since the previous November [1, 2]. Starting from February 2020, the various European countries have been hit by the COVID-19 outbreak at different degrees of severity and in different time frames. By mid-March, Europe was considered the active centre of the pandemic by the World Health Organization (WHO), with the number of new cases higher than in China. As social distancing is the most efficient way of containing the spread of the virus, "lockdown" measures, i.e., restricting people to their own homes, have been enforced across Europe [3], even if the combinations of actual control measures and the level of adherence to government recommendations have varied among countries [4]. With most of the population working from home, and school, non-essential commercial and productive activities closed, non-essential travel, and large gatherings of people stopped, we witnessed the biggest experiment ever conducted in Europe to assess the impact of a massive switch-off of atmospheric pollutant sources, especially road traffic [5]. However, some specific activities defined as essential, such as those linked to the agri-food industry, did not see any significant reduction.

Immediately after the lockdown enforcement, a dramatic decrease in nitrogen dioxide (NO_2_) concentrations over several European cities was detected by the Copernicus Sentinel-5P satellite. The European Space Agency (ESA) estimated that NO_2_ concentrations between March 13 and April 13, 2020, were 45 to 54% lower compared to the same period in 2019 in Europe [6].

Given the impact of the urban air quality on the European population’s health, this study focuses on in situ concentration data of the primary atmospheric pollutants measured in twelve European cities, characterised by different climatology and different emission sources and strengths. This is particularly important considering the complexity of atmospheric processes and chemical transformations involved in the formation of secondary pollutants, such as particulate matter (PM) and ozone (O_3_). Near real-time (NRT) daily concentration data of selected pollutants provided by the European Environment Agency (EEA) in the period from January 1 to June 30, 2020, have been considered, thus including in the analysis periods characterized by different degrees of restrictions.

Given the crucial role of meteorological parameters in determining pollutant dispersion, we include in our investigation a meteorological reanalysis providing a comprehensive description of meteorology and its evolution in the last five years (2016–2020). Here, we process precipitation, wind speed, and solar radiation data from ERA5 reanalysis produced by the European Centre for Medium-Range Weather Forecasts (ECMWF) [7]. Furthermore, to assess to what extent lockdown measures have impacted the composition of the selected cities’ atmosphere, we compare the daily average pollutant concentration data (PM_10_, PM_2.5_, NO_2_, O_3_) observed during the last four-year term, Jan-Jun 2016–2019. Finally, we consider the differences in lockdown measures across the European countries using the Oxford COVID-19 Government Response Tracker (OxCGRT). OxCGRT systematically collects information on several common policies that governments adopted to respond to the pandemic [8]. This unique circumstance allows us to understand to what extent the various emission sectors are contributing to urban air pollution and quantify the role of the specific sectors in determining the measured levels of pollutants. This role so far has been assessed only by air quality models. Several studies have been released describing the impact of lockdown on pollutant levels in Europe [e.g., 9–16]. Many of these studies compare the differences in concentration values, also considering the role of meteorology in determining the dispersion of pollutants.

**Fig 1:**
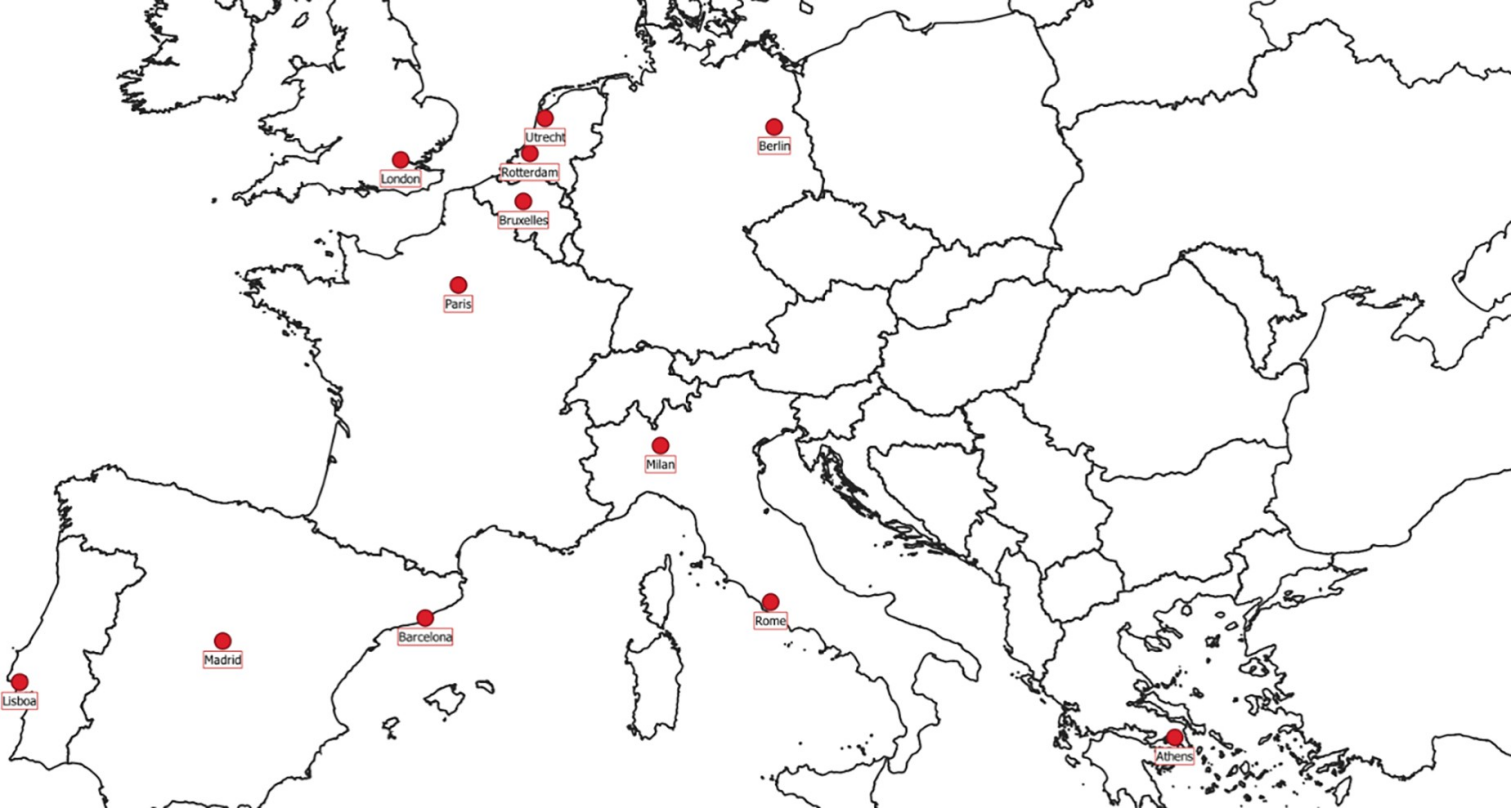
Study domain. Map showing the cities examined in the study.

In this paper, we propose a methodology for the fair comparison of lockdown impacts in twelve European cities (Athens, Barcelona, Berlin, Brussels, Lisbon, London, Madrid, Milan, Paris, Rome, Rotterdam, and Utrecht, see Fig 1). We explicitly consider the non-stationarity of meteorological conditions and emissions, which are progressively declining due to the adoption of stricter air quality measures.

The cities considered represent about 35 million inhabitants or 10% of the European urban population and span an area extending between 37° and 53° N and between 9°W and 23°E. The analysis is based on a common specifically-designed database (meteorology, pollution, and Covid-19 restriction levels) available for all the cities from the same data sources (S1–S4 Tables).

Such analysis is of paramount importance in designing air quality policies to effectively tackle air pollution in European cities.

## Materials and methods

The data source for ambient pollutant levels is the COVID-19 European Environment Agency (EEA) database reporting daily mean concentrations of three pollutants (PM_10_, PM_2.5_, NO_2_) in urban-traffic and urban-background stations from January 1, 2016, to June 30, 2020 [11].

All data are publicly available (see S1 Table for the links to the data repositories). Maximum daily 8-hour (max-8h) O_3_ concentrations, not included in the COVID-19 database, are based on data from the EEA air quality repository [17] (see S1 Table). The choice of using a common database allowed us to have homogeneous coverage over the twelve cities included in this study. However, this choice has limited the analysis to the daily average. Moreover, some parameters were missing in the selected cities: PM_2.5_ data are not reported for Barcelona, Berlin, and Paris (see S2 Table).

The Meteorological database has been populated with the daily mean values of the variables (10m u and v components of wind, total accumulated precipitation, surface net solar radiation, 2m surface temperature) simulated and reanalysed by ERA5-Land from January 1, 2016, to June 30, 2020 [18]. Links to the data repositories are given in S3 Table.

The COVID-19 Government Response Stringency Index (OxSI) is an aggregate measure of COVID-19 restrictions in European countries estimated by OxCGRT (https://covidtracker.bsg.ox.ac.uk, see also S4 Table).

To highlight the influence of lockdown restrictions on daily mean PM_10_, PM_2.5_, NO_2,_ and daily max8h O_3_, data observed during January-June 2020 are compared with those collected in 2016–2019 during the same period.

We assume that pollutant concentrations monitored in the spring of 2020 are affected by three processes: (i) the underlying emission trend due to the implementation of air quality EU and national policies, (ii) the meteorological conditions, and (iii) the emission reduction deriving from the lockdown restrictions.

To detect and identify these processes, the following methodology is designed and implemented for each city:

1. The lockdown period is determined based on the COVID-19 Oxford Stringency Index;
2. The trends in concentrations of pollutants are computed;
3. The meteorological conditions are classified;
4. The pollutant concentration variations due to COVID-19 restriction are analysed.

The above steps are detailed in the following sections.

### COVID-19 restriction classification

OxSI is a baseline measure of variation in governments’ responses. This composite measure is a simple additive score of seven indicators measured on an ordinal scale. The level of such indicators (varying from 0 to 100) accounts for the extent of COVID restriction implementation. OxSI is for comparative purposes only and should not be interpreted as a rating of the appropriateness or effectiveness of a country’s response.

In all countries, the COVID-19 restriction measures implied mainly a reduction in vehicular traffic fluxes. For instance, according to Google Traffic data [19], the decrease in retail and recreation, and work trips in March-May 2020 was around 50% in Belgium, 70% in the region around Milan, Italy, and 20% around Berlin, Germany, in comparison to previous years. However, these traffic data just represent variations with respect to a reference value and present strong daily fluctuations including a clear week periodicity. Thus, they were not used in the present study.

In January-June 2020, we identify three time periods:

- Pre-lockdown (preLD): days before the first pandemic wave characterized by a value of the OxSI lower than 70
- Lockdown (LD): days with significant COVID restrictions (OxSI equal or higher than 70)
- Post-lockdown (postLD): days after the first pandemic wave characterized by a value of the OxSIlower than 70.
- The postLD did not occur during the considered period in Lisbon and London.

**Fig 2:**
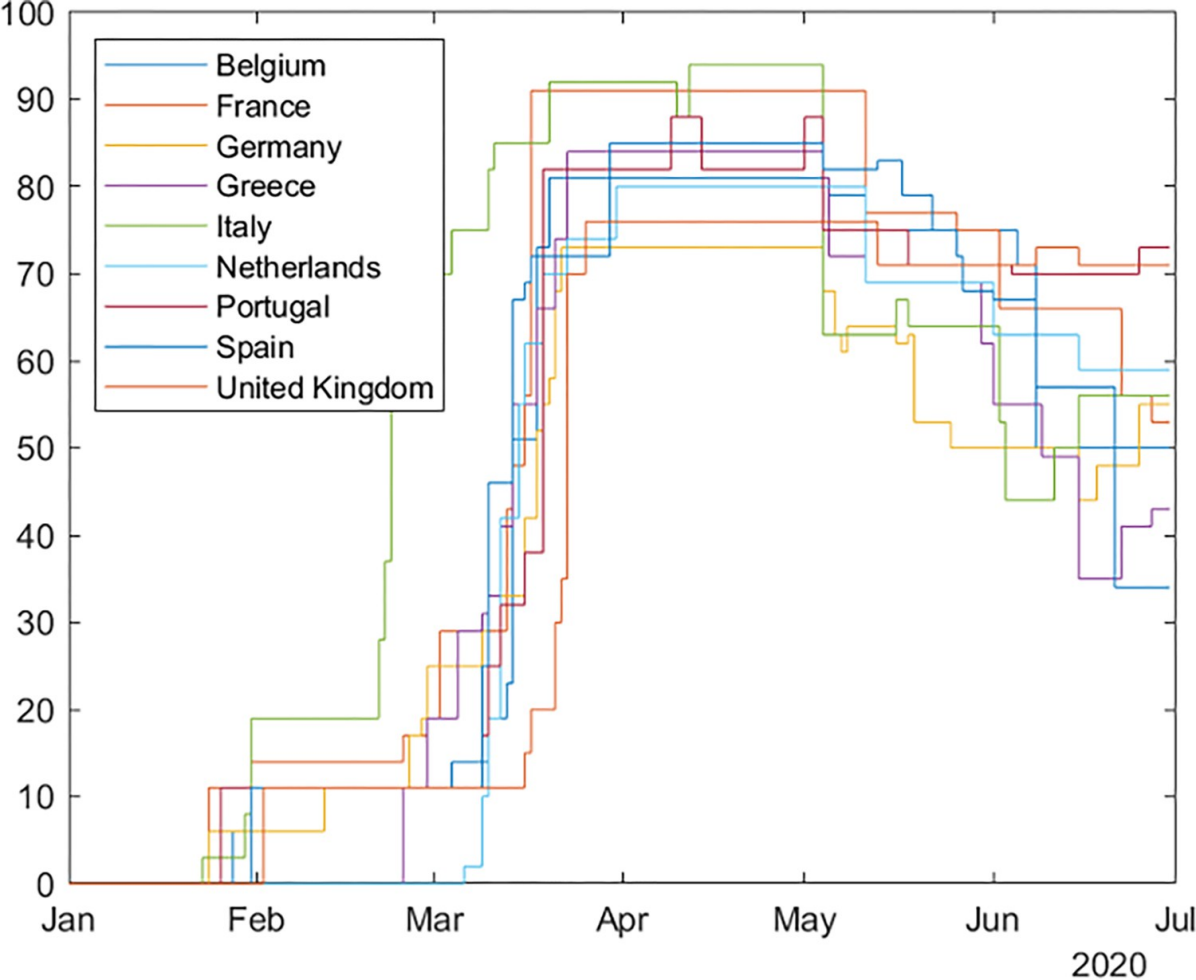
Oxford Stringency Index. Evolution of the Oxford Stringency Index in selected European countries.

Fig 2 shows the OxSI temporal evolution in the countries involved in this study. All countries reach the 70 threshold between March 15 and April 1. However, the number of days included in the lockdown period LD varies among the different cities. Additionally, an index value of 70 does not mean that the same type of restrictions is implemented everywhere (see the supplementary material for the calculation of OxSI), and even when the restrictions are similar, they may have been complied with by the population to a different extent. Finally, during the lockdown period, the index varies, reaching peak values above 90 in some countries such as Italy.

### Emission and concentration trends

Most of the cities considered in this study are characterized by air pollutant concentrations decreasing in time, following the application of increasingly stringent air quality policies. This requires discriminating between the 2020 concentration reductions caused by COVID-19 restrictions and the long-term decreasing trend due to the implementation of air quality policies.

A linear regression approach is applied to remove from the analysis the decreasing trend due to the implementation of air quality policies. The regression is performed using 2016 to 2019 concentration data derived from traffic and urban background monitoring stations for each pollutant and city. Trends are extrapolated to 2020 concentrations by adding the difference between the estimated concentrations on day *1* (2016-01-01) and the estimated concentrations on day *t* to the concentrations measured on day *t*. Given the generally decreasing concentration trends, this operation resulted in a systematic increase of adjusted values from January 2, 2016.

### Meteorological conditions and concentration clusters

To correctly assess the differences in the pollutant levels between 2020 and the preceding years, it is necessary to consider the role of meteorology. In this way, we avoid another possible misinterpretation of low concentration values in 2020, possibly due to a higher frequency of dispersive conditions. To this aim, we clustered days based on three meteorological variables measured as daily averages: (i) rainfall, (ii) wind speed, and (iii) surface net solar radiation, according to the following limits:

- rainy days: days with total accumulated precipitation higher than 2 mm^.^day^-1^;
- daily mean wind intensity (WI): weak wind below 2.5 m^.^s^-1^, medium wind between 2.5 and 5 m^.^s^-1^, and intense wind above 5 m^.^s^-1^;
- daily mean net solar radiation (SR): low under 120 W^.^m^-2^, moderate between 120 and 240 W^.^m^-2^, and strong above 240 W^.^m^-2^.

The wind intensity and solar radiation thresholds allow identifying nine different classes of non-rainy days, which can be grouped into "dispersive" and "non-dispersive" meteorological conditions, as shown in Table 1.

**Table 1:** Meteorological conditions. Features describing dispersive and non-dispersive conditions.

		Net Radiation W^.^m^-2^		
		SR < 120	120≤ SR < 240	240 ≤ SR
Wind Intensity m^.^s^-1^	WI < 2.5	Non-dispersive	Non-dispersive	Dispersive
	2.5 ≤ WI < 5.0	Non-dispersive	Dispersive	Dispersive
	5.0 ≤ WI	Dispersive	Dispersive	Dispersive

To summarize, the data set has been divided into rainy and not-rainy days, with the latter being clustered into "dispersive" and "non-dispersive."

### Analysis of pollutant concentrations due to COVID-19

**Fig 3:**
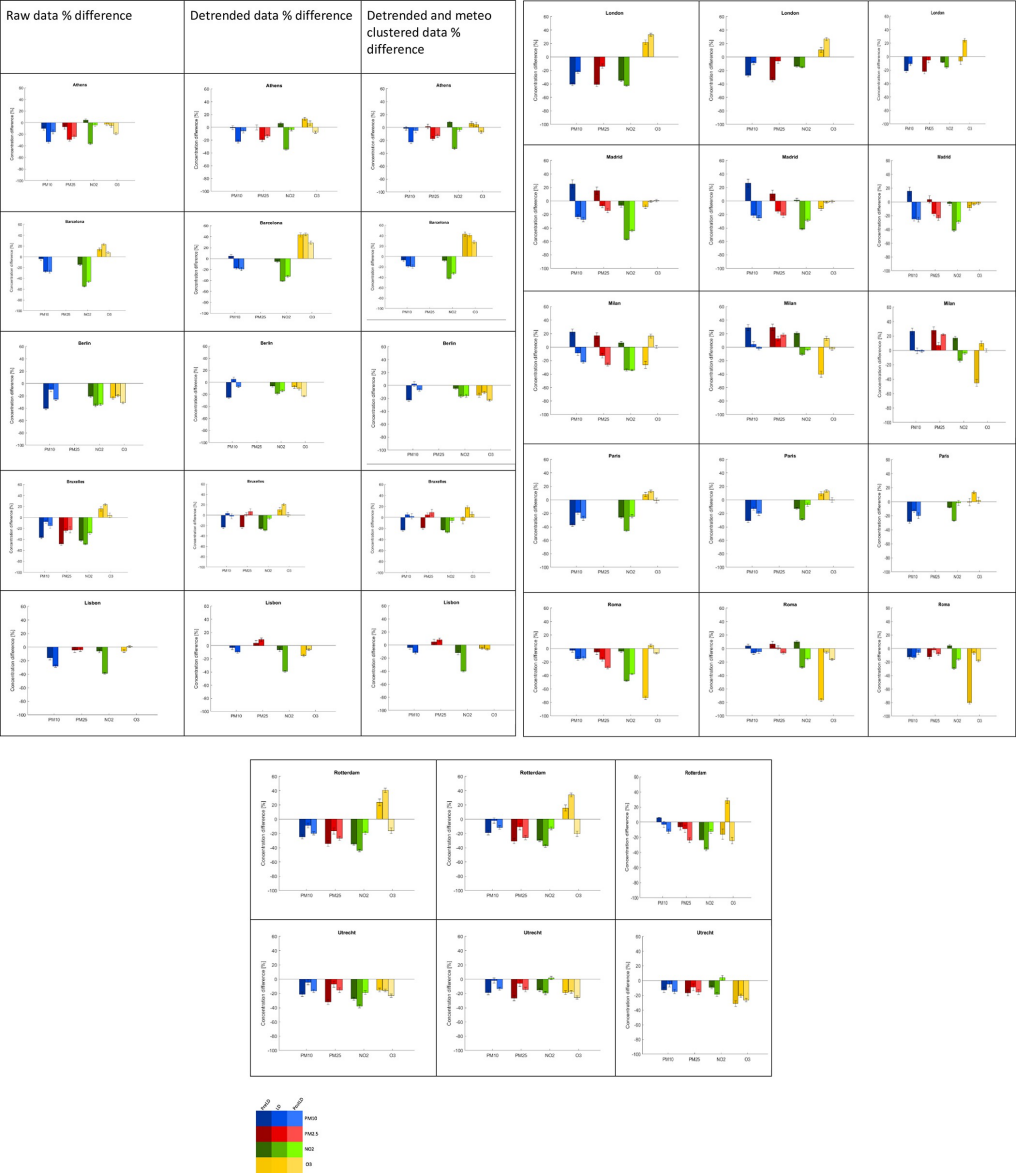
Results. Percentage changes of PM_10_ (blue), PM_2.5_ (red), NO_2_ (green), and O_3_ (yellow) of daily mean concentrations during preLD (dark bars), LD (medium bars), and postLD (light bars) periods in 12 European cities. Column 1: raw data, no processing applied; column 2: detrending only applied; column 3: detrending and meteorological clustering applied.

Combining the meteorological conditions and COVID-19 restriction classification, the resulting frequencies of occurrence (%) for the considered cities are reported in Fig 3. Notable differences immediately emerge between cities like Lisbon, where dispersive conditions are always prevalent, and other cities, for instance, Milan, where non-dispersive conditions represent the majority of the occurrences. Significant differences also emerge in some cases between meteorological conditions in 2020 and the corresponding periods in 2016–2019. In Paris, for example, non-dispersive conditions were present 36% of the days in 2016–2019 against only 27% in 2020. Similarly, Rotterdam had 32% rainy days with 42% of dispersive conditions in 2016–2019 and 26% of rains with 62% dispersive days in 2020.

The presence of precipitation strongly affects mean pollutant concentrations on rainy days. Therefore, we do not include rainy days in the comparison between 2020 and the preceding years.

Concentrations measured in 2020 and classified in each meteorological category are averaged for the defined periods: pre-lockdown (preLD20), lockdown (LD20), post-lockdown (postLD20). We compare these concentrations to those occurring at the corresponding dates in the previous years, to which we refer as LD16-19, preLD16-19, and postLD16-19, respectively.

The meteorological classes have a different frequency of occurrence in the two considered periods (2016–2019 and 2020). We thus weigh the average daily detrended concentrations related to the 2020 meteorological classes with the frequency of occurrence of the 2016–2019 classes. After doing so, we assess the difference in percent concentrations between the two periods.

Such a statistical approach aims at normalising meteorological conditions in 2020 to those of 2016–2019. However, it cannot take into account possible impacts on pollutant levels due to atmospheric dynamics. For instance, several consecutive days characterised by non-dispersive conditions lead to higher pollution levels than the same number of non-dispersive conditions for non-consecutive days. The described steps make the comparison between the first six months of 2020 and the previous years more robust.

## Results

We assess the impact of COVID restriction measures on pollutants’ levels, considering the long-term effect of AQ policies and the contingent effect of meteorological conditions. In Fig 3, we report the percent difference between the pollutants’ averaged concentrations during preLD, LD, and postLD (dark, medium, light bars) in 2020 and 2016–2019 for PM_10_, PM_2.5_, NO_2,_ and O_3_ (blue, red, green, yellow bars, respectively).

The pollutant concentration percentage changes are shown for

- column 1: raw data, no processing applied;
- column 2: detrending only applied;
- column 3: detrending and meteo clustering applied.

Column 1 shows differences between average pollutant concentration levels recorded in 2020 and 2016–2019 during the three considered periods. PM_10_, PM_2.5,_ and NO_2_ all show negative variation during lockdown and post-lockdown (when some restrictions were still in place). The straightforward message is that, if we consider the raw data, COVID-19-related restrictions have decreased these pollutant concentrations in all the cities considered in this study. However, such a conclusion is simplistic since it does not consider the long-term effects of the implementation of AQ policies in Europe (column 2) and the role of meteorology (column 3).

In column 2, we observe the pollutant concentration variation that would have occurred if the 2016–2019 trend was maintained with no emission perturbation due to COVID-19 restrictions in 2020.

For example, Athens shows that percent differences in PM10 levels during LD go from 32 to 22% for raw and detrended data. This suggests that the 22% difference is attributable to the reduced emission and meteorological conditions during LD20, whereas the remaining 10% might be ascribable to AQ policies.

In general, when variations in column 2 are smaller than those in column 1 (as in most of the cases), this means that concentration levels decreased more than would be expected considering only the long-term trend, suggesting either a decrease in emissions or the occurrence of meteorological conditions more favourable to pollutant dispersion, with respect to the previous four years.

When variations in column 2 are more significant than those in column 1, it means that concentrations did not decrease as much as expected when also taking into account the long-term decreasing trend. This suggests either an increase in emissions (not likely during LD and post-LD) or the occurrence of meteorological conditions less favourable to pollutant dispersion compared to the previous four years.

Column 3 refers to concentration levels that would have occurred in 2020 if 2020 had had the same dispersive condition (number of dispersive and non-dispersive days) as 2016–2019.

The comparison between columns 2 and 3 shows, in general, the same behaviour in all cities and gives a measure of where and to what extent COVID-19-related restrictions are the main drivers of changes in urban atmospheric composition.

The overall view that emerges from Fig 3 is quite complex and diverse for the various pollutants.

A more detailed description of the frequency of occurrence of meteorological conditions and the impact on pollutants’ atmospheric levels for all cities, is given in S1 and S2 Figs, respectively.

### Particulate matter

PM shows a diverse behaviour in the 12 cities due to the complex underlying dynamics, such as long-range transport and secondary aerosol formation processes.

In most cities, in column 3 of Fig 3, PM_10_ and PM_2.5_ variations during the lockdown are negative, meaning that the emission reductions due to COVID-19-related restrictions have determined a decrease in concentrations beyond the trend and regardless of meteorological conditions.

In the following, we analyse exceptions to this general behaviour.

**Fig 4:**
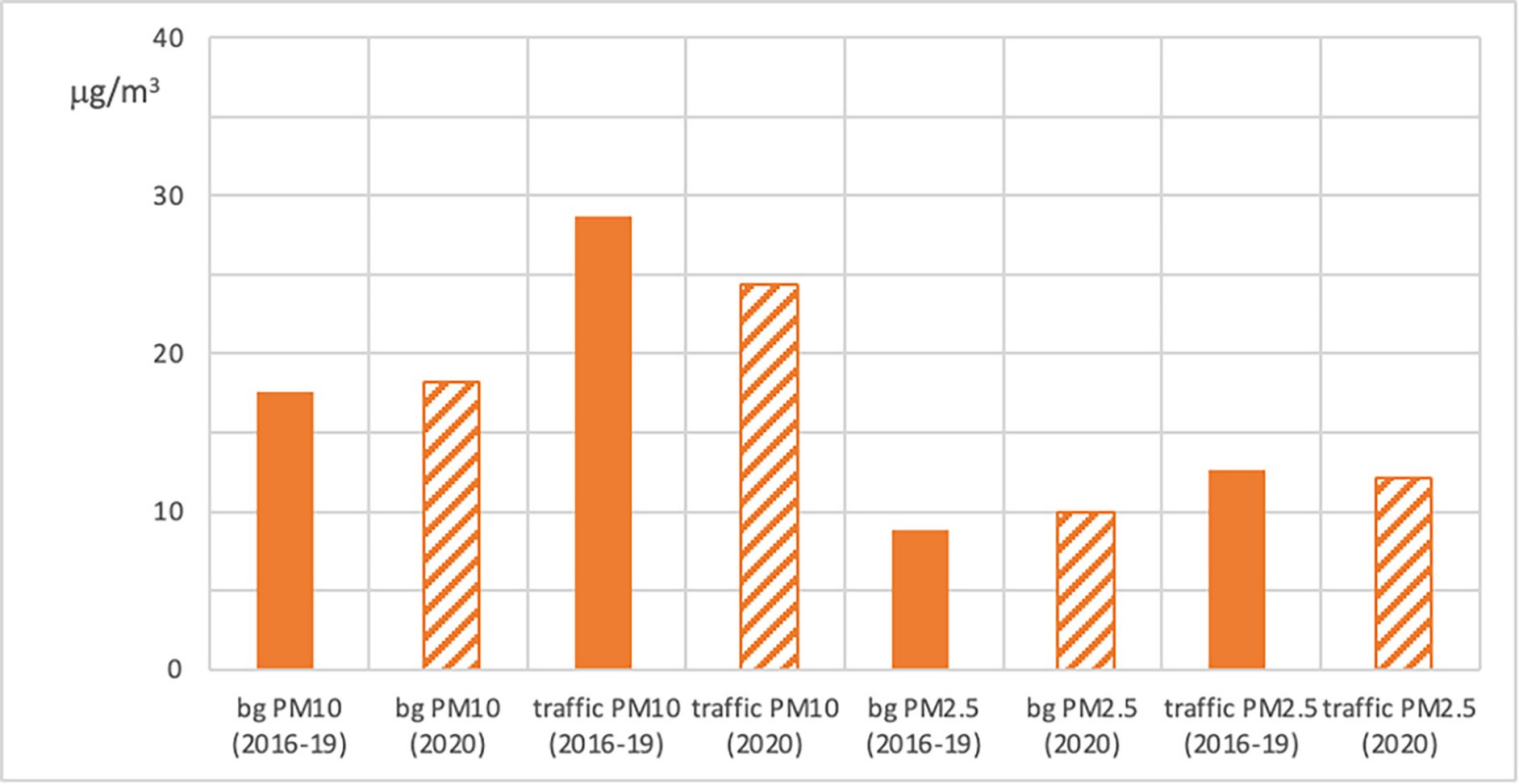
PM in Lisbon. PM_2.5_ and PM_10_ concentrations [μg^.^m^-3^] in background (bg) and traffic stations in Lisbon during LD.

In Lisbon, PM_10_ and PM_2.5_ variations during LD are decoupled with a decrease of about 10% in PM_10_ and an increase of about 7% in PM_2.5_ with respect to 2016–2019. Here, even if the percentage difference between PM_10_ and PM_2.5_ is not negligible, it must be noted that the absolute difference is about a 1μg/m^3^ increase for PM_2.5_ and a 2.5μg/m^3^ decrease for PM_10_. The increase in PM_2.5_ during LD, also in dispersive conditions, is shown in Fig 4, which depicts a more detailed representation of PM_2.5_ and PM_10_ concentration data in Lisbon. In traffic monitoring stations, both PM_2.5_ and PM_10_ concentrations benefit from the substantial reduction of urban traffic during LD [20]. This is not the case in background stations, where a slight increase of PM_2.5_, whose levels mainly result from secondary processes occurring in the larger city domain, is observed in LD20 compared to previous years.

In Brussels, where only background data are available for PM, a slight PM concentration increase is observed during LD. This could be explained by a ventilation factor (estimated as wind speed per boundary layer height) lower than usual, as reported by IRCELINE [21], suggesting that the role of meteorology is prevailing in emission reduction.

The Milan case is more complex. January and February 2020 (pre-LD-2020) are characterized by abnormally high levels of PM_10_ and PM_2.5_ (above the average 2016–2019) due to adverse meteorological conditions, i.e., persistent non-dispersive conditions during the whole period (see S1 Fig).

As the COVID-19 restrictions come into place, the concentration variations with respect to 2016–2019 become negative (column 1, Fig 3). This is also maintained in the post lockdown when restrictions are still in place. Beyond this first analysis, when looking at the detrended differences during lockdown and post-lockdown (column 2, Fig 3), we observe that variations are positive for PM_2.5_ and close to zero for PM_10_.

Standardizing the meteorological condition (column 3, Fig 3), the PM_10_ percentage variation equals zero, while PM_2.5_ concentration variations remain positive. This analysis suggests that PM_2.5_ concentrations, despite the COVID-19 restrictions, are higher than expected, assuming the implementation of AQ directives and plans. This might be due to the non-linearity of the PM fine fraction formation processes involving atmospheric NH_3_ [22]. This conclusion is supported by the finding that NH_3_ concentration levels in Lombardy during the lockdown increased compared to previous years [23].

As PM_2.5_ concentration variation is positive and PM_10_ one is zero, we infer that, during the lockdown, the PM coarse fraction decreases mainly due to traffic-related fragmentation and resuspension processes.

### Nitrogen dioxide

NO_2_ concentration variations during LD are negative, suggesting that its concentrations are lower than expected, assuming only the implementation of AQ measures. This behaviour is consistent with COVID-19 restriction enactment and suggests that AQ policies are effective for such pollutants.

The NO_2_ concentration reduction is fairly evident in both dispersive and non-dispersive conditions in all cities, as shown by satellite observations [6, 24].

This suggests that the emission abatement due to the COVID-19 restrictions is the primary driver of change. Furthermore, such a reduction is consistent with vehicular traffic reduction, the sector most affected by COVID-19 restrictions, being the primary source of NOx in urban areas.

The NO_2_ variations range from 15% (Berlin, London, Milan) to 40% (Barcelona, Lisbon, Madrid) compared to the 2016–2019 average. Such differences in percentage reduction are likely to be linked to different emission mixes in the various cities.

We can therefore assume that a low emission traffic policy might be an effective measure to reduce population exposure to NO_2_, even if to a different extent across European cities.

### Tropospheric ozone

Similarly, to secondary PM, O_3_, a photochemical pollutant whose dynamics are not linear [25], presents a complex behaviour, as shown in Fig 3.

Looking at the differences between columns 1 and 2 in Athens, Barcelona, Brussels, London, Paris, and Rotterdam, AQ measures seem to have been ineffective in reducing O_3_ levels.

Standardizing the meteorological condition to 2016–2019, during LD20 in some cities (Brussels, London, Milan, Paris, Rotterdam), O_3_ shows a clear increase in correspondence of NO_2_ reductions >15% in both dispersive and non-dispersive meteorological conditions (see Supplementary Material), showing the typical behaviour of a VOC-limited regime. Similar conclusions have been drawn by other authors [26, 27].

## Conclusions

The COVID-19 restrictions applied almost simultaneously in March-June 2020 to the wide European domain represent an unprecedented experiment. The analysis of pollutant levels allowed us to assess the impact on urban air quality of measures based mainly on traffic limitations, having been other sectors, especially domestic heating, and agriculture, not significantly affected by the pandemic restrictions.

Altogether, analysing these unique circumstances allowed us to estimate the impact of a nearly zero-emission urban transport scenario in 12 European cities.

We compared the pollutant levels monitored during the first six months of 2020 with the average concentrations observed in the same period of the previous four years (2016–2019), adopting a methodology that considers long-term trends and natural variability. The detrending analysis rules out the impact of EU and national air quality policies. At the same time, the study of the different meteorological conditions suggests where the emission reduction due to the COVID-19 restrictions is effective regardless of local specific feature

The analysis shows that PM concentrations in each city can respond differently to the same emission reduction measure. This happens because other non-controlled emissions vary from city to city (for instance, when rural areas and agricultural activities surround the city) and/or because the local meteorology significantly interferes with the transformation processes of precursors into fine particles.

The most evident result, common to all the cities, is that a dramatic traffic reduction effectively reduces NO_2_ concentrations, also considering the AQ policies and the natural variability impacts. However, how NO_2_ concentration reduction is affecting PM and O_3_ levels is hard to predict due to the non-linearity of the underlying chemical processes.

In a VOC-limited regime, O_3_ concentrations increase as expected, highlighting that a plan targeting mainly road traffic cannot be generally considered an effective policy for abating both pollutants, NO_2_ and O_3_.

From the policy point of view, these findings suggest that Europe-wide emission measures targeting urban traffic, even when representing the so-called best practices, may not be the only practical option. Regional/local integrated assessment models that consider each urban context’s peculiarities are, on the contrary, a more appropriate approach to drive multi-scale policies aiming at improving air quality. Such models can also assess not only the effectiveness but also the efficiency of a set of measures by estimating their local implementation costs, the impacts on population and ecosystem health, as well as GHGs emissions.

## Supporting information

S1 Fig Meteorological classes. Frequencies of occurrence (%) of meteorological classes in 2016–2019 and 2020, during the lockdown and no-lockdown periods.
(PDF)

S2 Fig Percentage concentration differences of pollutants under different conditions. Percentage changes of PM_10_ (blue), PM_2.5_ (red), NO_2_ (green), and O_3_ (yellow) of daily mean concentrations. Darker to lighter: dispersive pre-lockdown, dispersive lockdown, dispersive post-lockdown, non-dispersive pre-lockdown, non-dispersive lockdown, non-dispersive post-lockdown.
(PDF)

S1 Table Concentration data. Links to PM_10_, PM_2.5_, NO_2_ and O_3_ concentration data repository for the period January 1—June 30 (2016–2020).
(DOCX)

S2 Table Concentration data availability. Green cells represent concentration data of PM_10_, PM _2.5_, NO_2_ and O_3_ available for the individual cities; red cells represent a lack of data.
(DOCX)

S3 Table ERA5-Land reanalysis. Meteo variables used in this study and link to the ERA5-Land Reanalysis for the period January 1—June 30 (2016–2020).
(DOCX)

S4 Table Oxford Stringency Index. OxSI (January 1—June 30, 2020) link to data source.
(DOCX)
