# Supplementary figures and images for: The greatest air quality experiment ever: Policy suggestions from the COVID-19 lockdown in twelve European cities
Source: PLoS One. 2022 Nov 30;17(11):e0277428. doi: 10.1371/journal.pone.0277428 (PMC9710802; doi:10.1371/journal.pone.0277428)

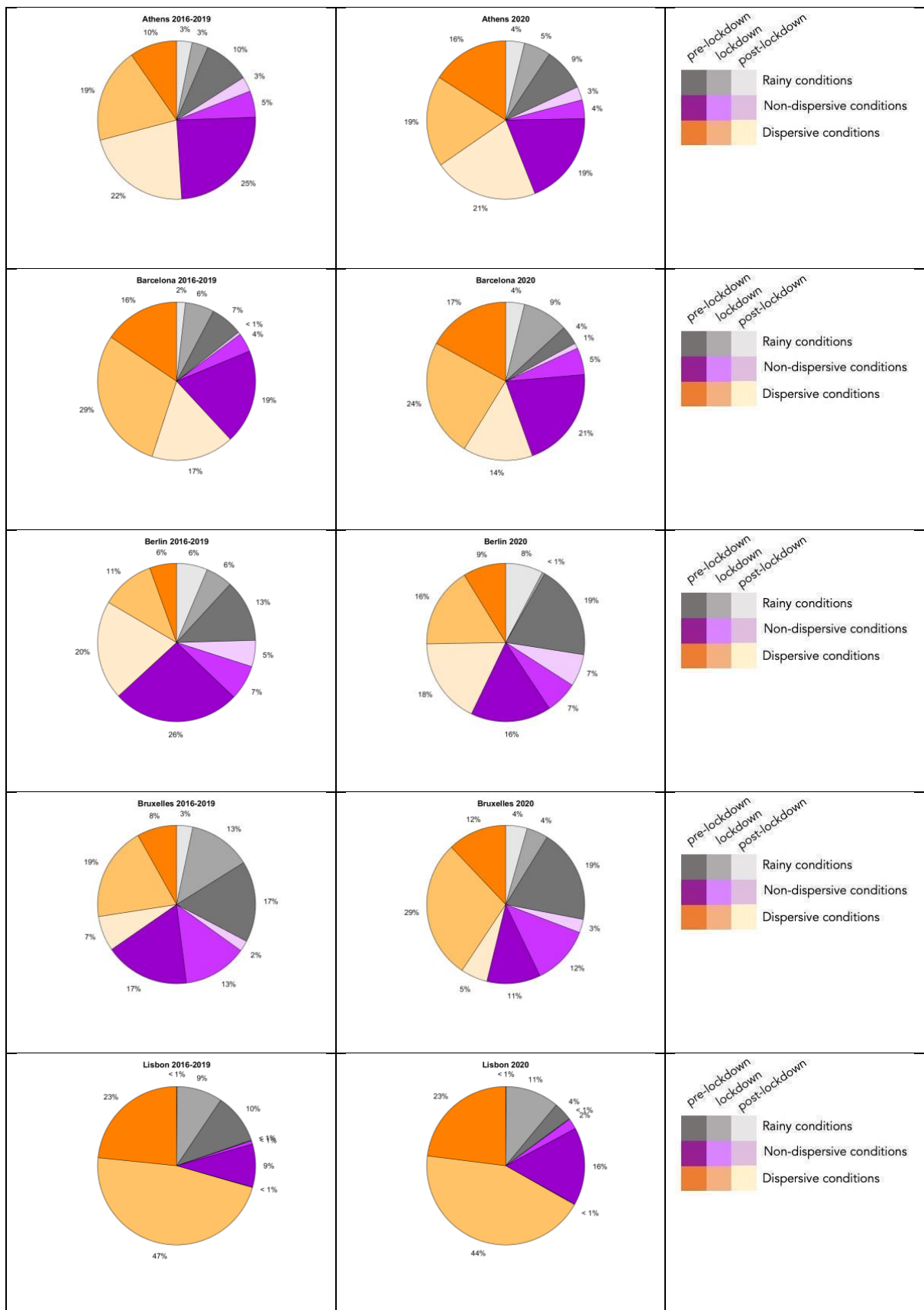

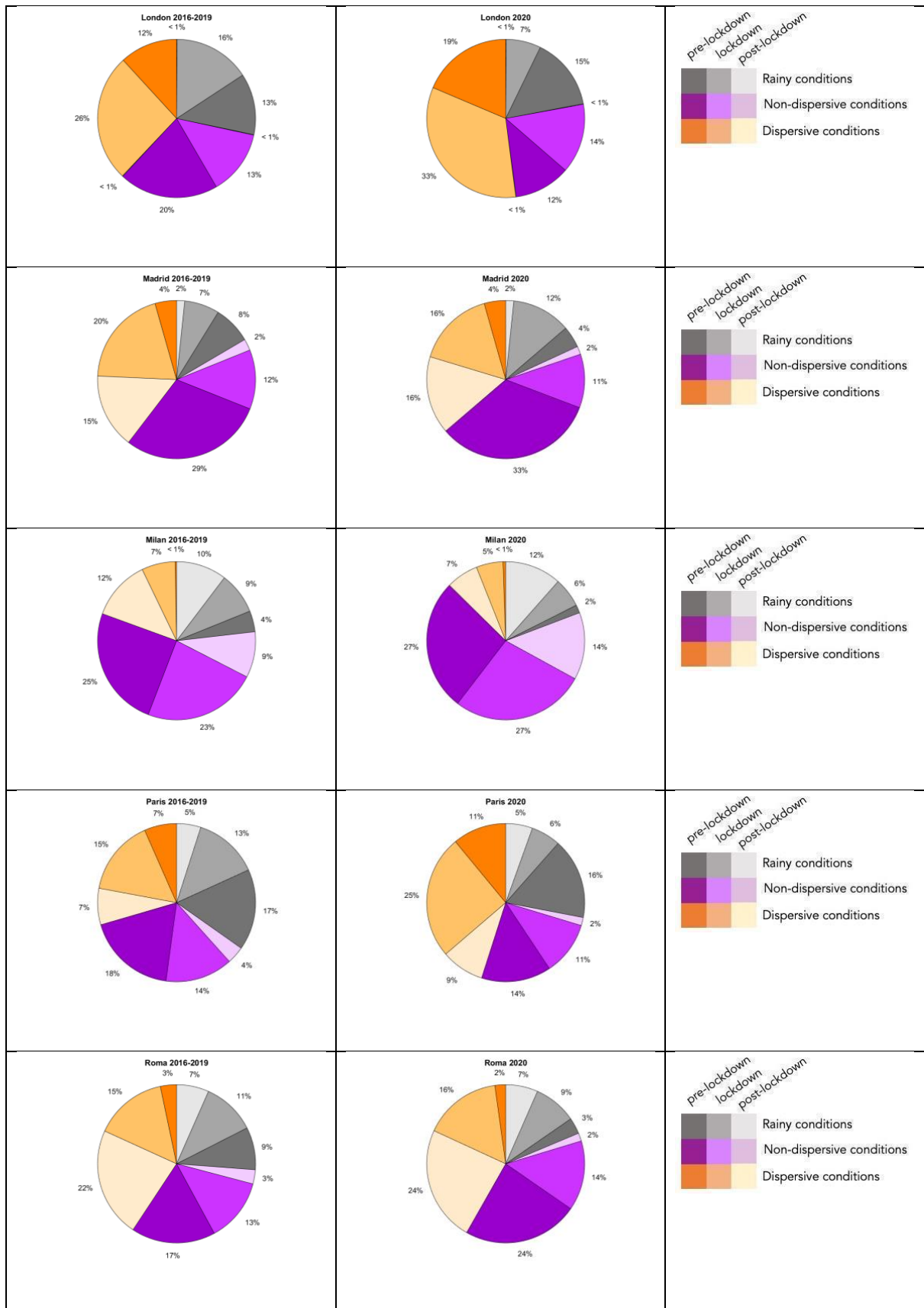

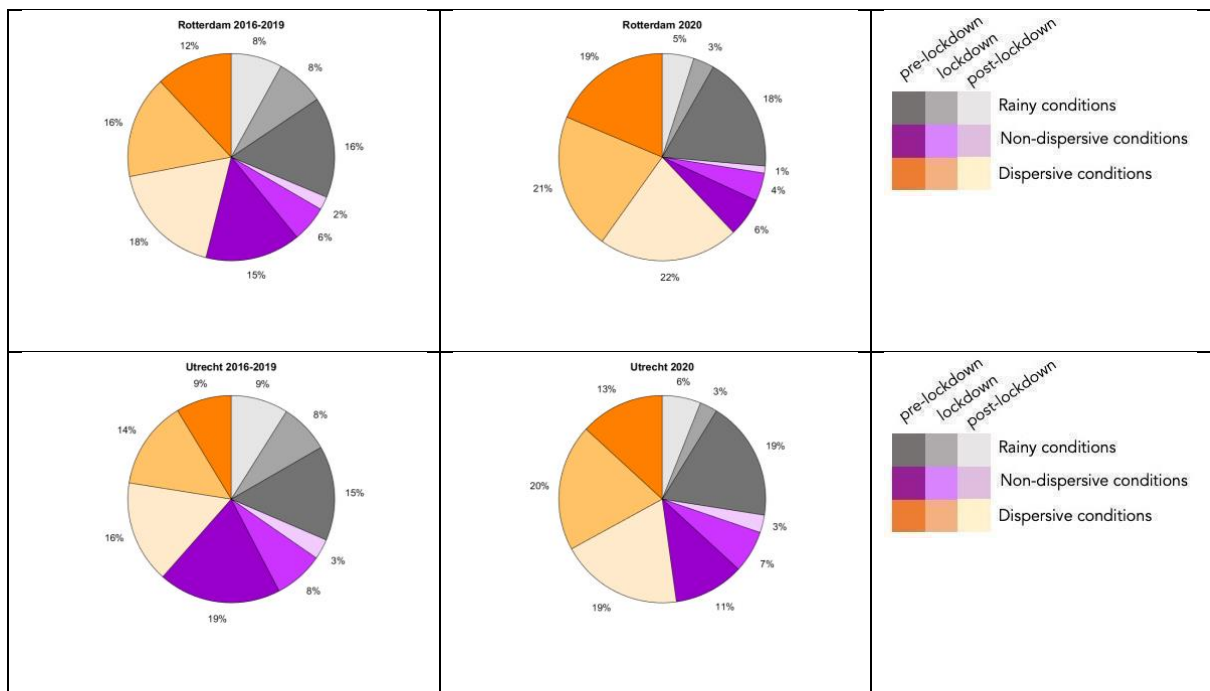

Supplement: S1 Fig — Frequencies of occurrence (%) of meteorological classes in 2016–2019 and 2020, during the lockdown and no-lockdown periods. (PDF) [file pone.0277428.s001.pdf]

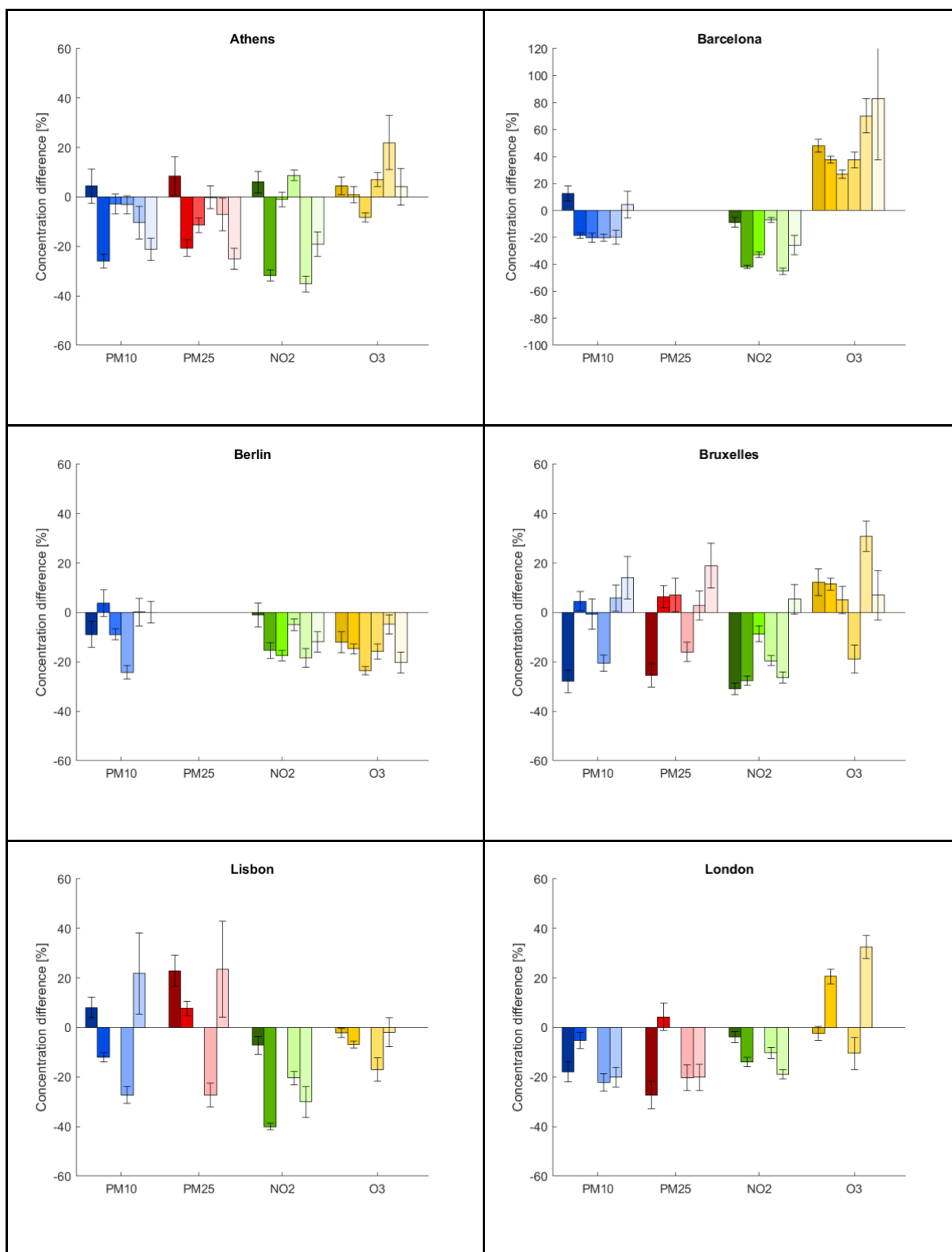

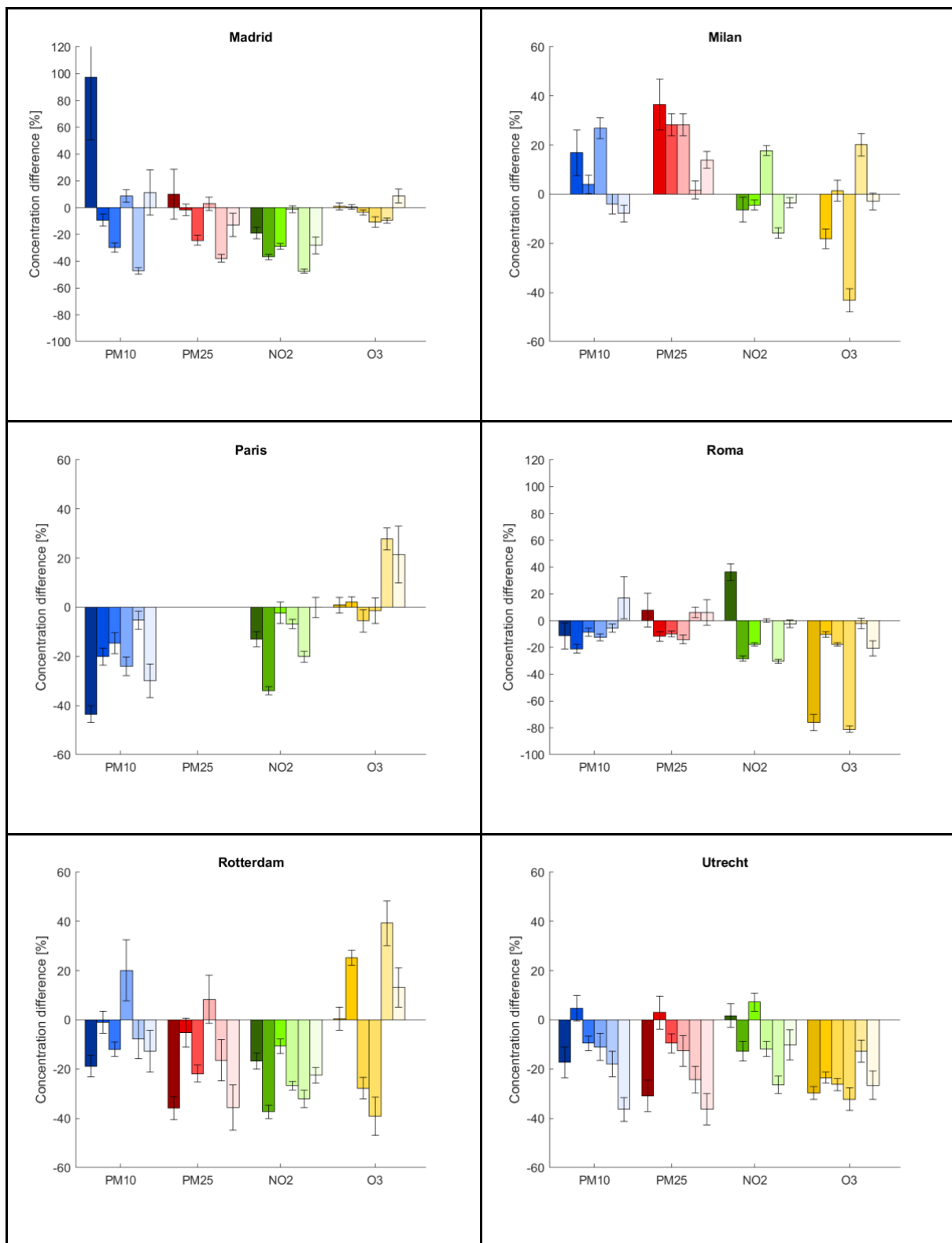

Supplement: S2 Fig — Percentage changes of PM10 (blue), PM2.5 (red), NO2 (green), and O3 (yellow) of daily mean concentrations. Darker to lighter: dispersive pre-lockdown, dispersive lockdown, dispersive post-lockdown, non-dispersive pre-lockdown, non-dispersive lockdown, non-dispersive post-lockdown. (PDF) [file pone.0277428.s002.pdf]
