# Supplementary material for: The greatest air quality experiment ever: Policy suggestions from the COVID-19 lockdown in twelve European cities
Source: PLoS One. 2022 Nov 30;17(11):e0277428. doi: 10.1371/journal.pone.0277428 (PMC9710802; doi:10.1371/journal.pone.0277428)
Supplement: S1 Table — Links to PM10, PM2.5, NO2 and O3 concentration data repository for the period January 1—June 30 (2016–2020). (DOCX) [file pone.0277428.s003.docx]

| EEA Concentration data | source |
| --- | --- |
| PM_10_ | <https://www.eea.europa.eu/themes/air/air-quality-and-covid19> |
| PM_2.5_ | <https://www.eea.europa.eu/themes/air/air-quality-and-covid19> |
| NO_2_ | <https://www.eea.europa.eu/themes/air/air-quality-and-covid19> |
| O_3_^a^ | <https://discomap.eea.europa.eu/map/fme/AirQualityExport.htm> |
