# Supplementary material for: The greatest air quality experiment ever: Policy suggestions from the COVID-19 lockdown in twelve European cities
Source: PLoS One. 2022 Nov 30;17(11):e0277428. doi: 10.1371/journal.pone.0277428 (PMC9710802; doi:10.1371/journal.pone.0277428)
Supplement: S2 Table — Green cells represent concentration data of PM10, PM 2.5, NO2 and O3 available for the individual cities; red cells represent a lack of data. (DOCX) [file pone.0277428.s004.docx]

|  |  |  | Athens | Barcelona | Berlin | Brussels | Lisbon | London | Madrid | Milan | Paris | Rome | Rotterdam | Utrecht |
| --- | --- | --- | --- | --- | --- | --- | --- | --- | --- | --- | --- | --- | --- | --- |
| NO_2_ | traffic | 2016 |  |  |  |  |  |  |  |  |  |  |  |  |
|  |  | 2017 |  |  |  |  |  |  |  |  |  |  |  |  |
|  |  | 2018 |  |  |  |  |  |  |  |  |  |  |  |  |
|  |  | 2019 |  |  |  |  |  |  |  |  |  |  |  |  |
|  |  | 2020 |  |  |  |  |  |  |  |  |  |  |  |  |
|  | background | 2016 |  |  |  |  |  |  |  |  |  |  |  |  |
|  |  | 2017 |  |  |  |  |  |  |  |  |  |  |  |  |
|  |  | 2018 |  |  |  |  |  |  |  |  |  |  |  |  |
|  |  | 2019 |  |  |  |  |  |  |  |  |  |  |  |  |
|  |  | 2020 |  |  |  |  |  |  |  |  |  |  |  |  |
| PM_10_ | traffic | 2016 |  |  |  |  |  |  |  |  |  |  |  |  |
|  |  | 2017 |  |  |  |  |  |  |  |  |  |  |  |  |
|  |  | 2018 |  |  |  |  |  |  |  |  |  |  |  |  |
|  |  | 2019 |  |  |  |  |  |  |  |  |  |  |  |  |
|  |  | 2020 |  |  |  |  |  |  |  |  |  |  |  |  |
|  | background | 2016 |  |  |  |  |  |  |  |  |  |  |  |  |
|  |  | 2017 |  |  |  |  |  |  |  |  |  |  |  |  |
|  |  | 2018 |  |  |  |  |  |  |  |  |  |  |  |  |
|  |  | 2019 |  |  |  |  |  |  |  |  |  |  |  |  |
|  |  | 2020 |  |  |  |  |  |  |  |  |  |  |  |  |
| PM_2.5_ | traffic | 2016 |  |  |  |  |  |  |  |  |  |  |  |  |
|  |  | 2017 |  |  |  |  |  |  |  |  |  |  |  |  |
|  |  | 2018 |  |  |  |  |  |  |  |  |  |  |  |  |
|  |  | 2019 |  |  |  |  |  |  |  |  |  |  |  |  |
|  |  | 2020 |  |  |  |  |  |  |  |  |  |  |  |  |
|  | background | 2016 |  |  |  |  |  |  |  |  |  |  |  |  |
|  |  | 2017 |  |  |  |  |  |  |  |  |  |  |  |  |
|  |  | 2018 |  |  |  |  |  |  |  |  |  |  |  |  |
|  |  | 2019 |  |  |  |  |  |  |  |  |  |  |  |  |
|  |  | 2020 |  |  |  |  |  |  |  |  |  |  |  |  |
| O_3_ | traffic | 2016 |  |  |  |  |  |  |  |  |  |  |  |  |
|  |  | 2017 |  |  |  |  |  |  |  |  |  |  |  |  |
|  |  | 2018 |  |  |  |  |  |  |  |  |  |  |  |  |
|  |  | 2019 |  |  |  |  |  |  |  |  |  |  |  |  |
|  |  | 2020 |  |  |  |  |  |  |  |  |  |  |  |  |
|  | background | 2016 |  |  |  |  |  |  |  |  |  |  |  |  |
|  |  | 2017 |  |  |  |  |  |  |  |  |  |  |  |  |
|  |  | 2018 |  |  |  |  |  |  |  |  |  |  |  |  |
|  |  | 2019 |  |  |  |  |  |  |  |  |  |  |  |  |
|  |  | 2020 |  |  |  |  |  |  |  |  |  |  |  |  |
