# Supplementary material for: The greatest air quality experiment ever: Policy suggestions from the COVID-19 lockdown in twelve European cities
Source: PLoS One. 2022 Nov 30;17(11):e0277428. doi: 10.1371/journal.pone.0277428 (PMC9710802; doi:10.1371/journal.pone.0277428)
Supplement: S3 Table — Meteo variables used in this study and link to the ERA5-Land Reanalysis for the period January 1—June 30 (2016–2020). (DOCX) [file pone.0277428.s005.docx]

| ERA5-Land reanalysis meteo data | Source | Documentation |
| --- | --- | --- |
| 10m u-component of wind | <https://cds.climate.copernicus.eu/cdsapp#!/dataset/reanalysis-era5-single-levels?tab=form> | <https://confluence.ecmwf.int/display/CKB/ERA5%3A+data+documentation> |
| 10m v-component of wind |  |  |
| Total precipitation |  |  |
| Surface net solar ^a^ radiation |  |  |
| 2m temperature |  |  |
