# Supplementary material for: The greatest air quality experiment ever: Policy suggestions from the COVID-19 lockdown in twelve European cities
Source: PLoS One. 2022 Nov 30;17(11):e0277428. doi: 10.1371/journal.pone.0277428 (PMC9710802; doi:10.1371/journal.pone.0277428)
Supplement: S4 Table — OxSI (January 1—June 30, 2020) link to data source. (DOCX) [file pone.0277428.s006.docx]

**S4 Table. Oxford Stringency Index.** OxSI (January 1 - June 30, 2020) link to data source**.**

| Lockdown measures | Source | Documentation/publication |
| --- | --- | --- |
| Oxford Stringency Index | https://raw.githubusercontent.com/OxCGRT/covid-policy-tracker/master/data/OxCGRT_latest.csv | Hale, T. et al. "Variation in Government Responses to COVID-19" Version 7.0. Blavatnik School of Government Working Paper. May 25, 2020.  https://www.bsg.ox.ac.uk/sites/default/files/2020-09/BSG-WP-2020-032-v7.0.pdf |
